# A novel cytogenetic abnormality t(7;8)(p11.2:q11.2) and a four-way Philadelphia translocation in an imatinib mesylate-resistant chronic myeloid leukemia patient

**DOI:** 10.3892/ol.2012.1037

**Published:** 2012-11-21

**Authors:** WALID AL-ACHKAR, ABDULMUNIM ALJAPAWE, SUHER ALMEDANI, THOMAS LIEHR, ABDULSAMAD WAFA

**Affiliations:** 1 Department of Molecular Biology and Biotechnology, Human Genetics Division, Damascus 6091, Syria;; 2 Flow-Cytometry Laboratory, Department of Molecular Biology and Biotechnology, Mammalians Biology Division, Atomic Energy Commission, Damascus 6091, Syria;; 3 Jena University Hospital, Institute of Human Genetics, Jena 07743, Germany

**Keywords:** chronic myeloid leukemia, four-way Philadelphia translocation, fluorescence *in situ* hybridization, array-proven multicolor banding, imatinib mesylate

## Abstract

Chronic myelogenous leukemia (CML) is characterized by the Philadelphia (Ph) chromosome, created by a reciprocal translocation t(9:22)(q34;q11) which forms the chimeric gene, BCR-ABL. Variant Ph chromosome translocations involving chromosomes other than 9 and 22 have been identified in 5–10% of CML cases. Four-way Ph chromosome translocations are an extremely rare event in myeloid malignancies and the phenotypic consequences of such rearrangements have not been investigated. Deletions in chromosome 9 are known to be associated with a poor prognosis. In the present study, a novel case of Ph chromosome-positive CML in blast crisis is reported. A four-way Ph translocation was identified, involving five chromosomal regions, 9p21, 9q34, 12p13.3, 20q11.2 and 22q11.2, as well as an unbalanced translocation, der(7)t(7;8)(p11.2;q11.2). Since the majority of CML cases are currently treated with imatinib, variant rearrangements in general have no specific prognostic significance, although the mechanisms involved in resistance to therapy have yet to be investigated. In the present case, multiple partial deletions, including ABL and ASS genes on chromosome 9, the region 7p11.2 to 7pter, 8q11.2 to 8pter and two regions on chromosome 12, were identified. An additional Ph chromosome was also detected. Immunophenotyping indicated that the patient had biphenotypic leukemia. The patient did not respond positively to imatinib chemotherapy and died for unknown reasons, one month after diagnosis. The underlying mechanisms and prognostic implications of these cytogenetic abnormalities are discussed.

## Introduction

Chronic myelogenous leukemia (CML) is a myeloproliferative disease characterized by the Philadelphia (Ph) chromosome. This chromosome is created by a reciprocal t(9:22) translocation which transfers the Abelson (ABL) oncogene on chromosome 9 to the breakpoint cluster region (BCR) of chromosome 22, resulting in the formation of a fused BCR/ABL gene (1). BCR/ABL produces an abnormal tyrosine kinase that causes aberrant myelopoiesis identified in CML. Variant Ph chromosome translocations involving one or more chromosome regions in addition to chromosomes 9 and 22 have been identified in 5–10% of CML patients (2). In these variants, four-way Ph chromosome translocations are rare (3). The prognostic significance of variant Ph chromosome CML remains unclear.

The progression of CML from the chronic phase (CP) to blast crisis (BC) is frequently associated with non-random secondary chromosomal aberrations, including +8, i(17q), +19 and an extra Ph chromosome (4).

Since tyrosine kinase activity is required for the transforming function of the BCR/ABL fusion protein, imatinib, a specific inhibitor of the kinase, is an effective treatment for CML patients. The 5-year estimated overall survival rate for patients who receive imatinib as initial therapy is 89%. This rate is higher than those reported in previous studies of CML treatment and only 7% of all patients progressed to the accelerated phase (AP) or BC (5). In a previous study, deletions on the derivative chromosome 9 [der(9)] were identified at a higher frequency in patients with variant Ph translocations than in those with classic Ph (45 and 17%, respectively) (6).

In the present study, a novel case of a Ph chromosome-positive CML in BC was identified, with a four-way rearrangement involving five chromosomal regions, 9p21, 9q34, 12p13.3, 20q11.2 and 22q11.2, an unbalanced translocation der(7)t(7;8) (p11.2;q11.2), deletions of ABL and argininosuccinate synthetase (ASS) genes at 9q34 on der(9), partial monosomies 8, 12 and an additional Ph chromosome. In addition, immunopheno-type analysis indicated biphenotypic leukemia.

## Materials and methods

### Case report

In April 2011, a 22-year-old female presented with a white blood cell count (WBC) of 97×10^9^ cells/l (neutrophils, 21; lymphocytes, 73; eosinophiles, 4; monocytes, 1; and basophiles, 1%). The platelet count was 268×10^9^ cells/l and the hemoglobin level was 9.1 g/dl. Physical examination revealed splenomegaly and weight loss was noted. Chromosome analysis using banding cytogenetics demonstrated a karyotype consistent with clinical diagnosis of a CML in CP. The patient was treated daily with Zyloric (300 mg) and hydroxyurea (500 mg) for four days. LDH was 1860 U/l (normal, <460 U/l) and serum alkaline phosphase was 348 U/l (normal, <232 U/l). In September 2011, the patient presented for the second time with a WBC count of 132.4×10^9^ cells/l (neutrophils, 1; lymphocytes, 40; and immature cells, 52%). Platelet count was 22×109/l and the hemoglobin level was 10 g/dl. Imatinib mesylate (400 mg/day) was administered for five months and following this period the described symptoms were not observed. In October 2011, the patient died for unknown reasons under treatment.

### Chromosome analysis

Chromosome analysis using GTG-banding was performed according to standard procedures (7) prior to chemotherapeutic treatment. A total of 20 metaphase cells derived from unstimulated bone marrow culture were analyzed. Karyotypes are described according to the International System for Human Cytogenetic Nomenclature (8).

### Molecular cytogenetics

Fluorescence *in situ* hybridization (FISH) using LSI BCR/ABL+9q34 three color dual fusion translocation probe (Abbott Molecular/Vysis, Des Plaines, IL, USA) and chromosome enumeration probe (CEP) for chromosome 9 (Abbott Molecular/Vysis) were applied according to the manufacturer’s instructions together with a whole chromosome painting (WCP) probe for chromosomes 7, 8, 9, 12, 20 and 22 (MetaSystems, Altlussheim, Germany) (7). FISH using the corresponding chromosome specific array-proven multicolor banding (aMCB) probe sets based on microdis-section derived region-specific libraries was performed as previously described (7). A minimum of 20 metaphase spreads were analyzed, using a fluorescence microscope (AxioImager.Z1 mot, Carl Zeiss Ltd., Hertfordshire, UK) equipped with appropriate filter sets to discriminate between a maximum of five fluorochromes and the counterstain DAPI (4′,6-diamino-2-phenylindole). Image capture and processing were performed using the ISIS imaging system (MetaSystems).

### Reverse transcription polymerase chain reaction (RT-PCR) for BCR/ABL fusion transcripts

Total RNA was extracted from the diagnostic peripheral blood sample using the InviTrap RNA kit (Invitek GmbH, Berlin, Germany) according to the manufacturer’s instructions. cDNA was prepared from 5μg total RNA with the Genequality BCR-ABL kit (AB Analitica, Padova, Italy) according to the manufacturer’s instructions.

### Flow cytometry immunophenotyping

Immunophenotyping of leukemic blasts was performed as previously described (7).

### DNA sequencing

Detection of BCR/ABL mutation domain was performed using previously described primers (9).

## Results

**Figure 1.**
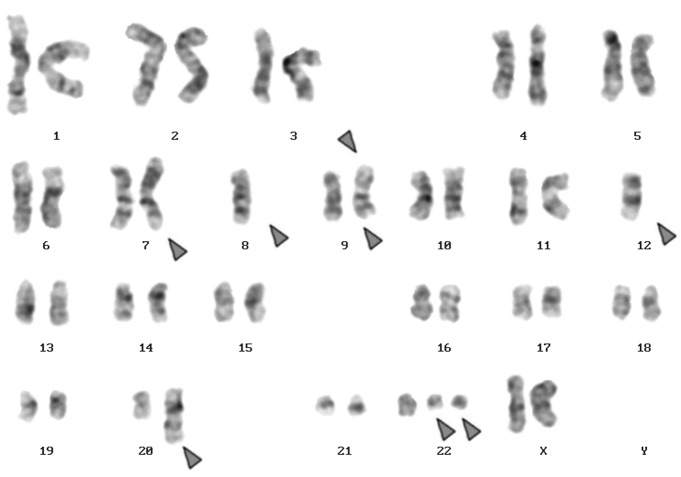
GTG-banding revealed a complex karyotype involving four further chromosomes, monosomies 8, 12 and an extra copy of the Ph chromosome in addition to chromosomes 9 and 22. All derivative chromosomes are high-lighted by arrow heads.

**Figure 2.**
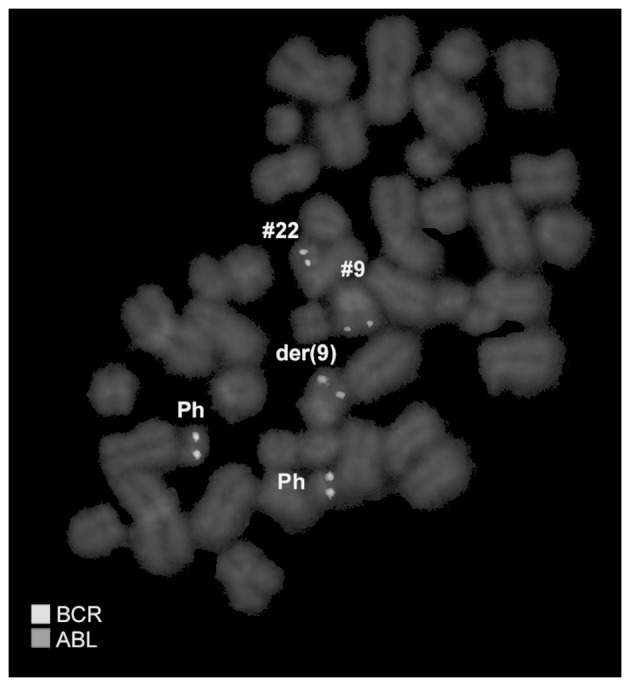
Metaphase FISH using probes for BCR (#22), ABL (#9) and ASS (#9) revealed two Ph chromosomes and absence of ABL and ASS genes on der(9). #, chromosome; der, derivative chromosome; Ph, Philadelphia; FISH, fluorescence *in situ* hybridization; BCR, breakpoint cluster region; ABL, Abelson; ASS, argininosuccinate synthetase.

**Figure 3.**
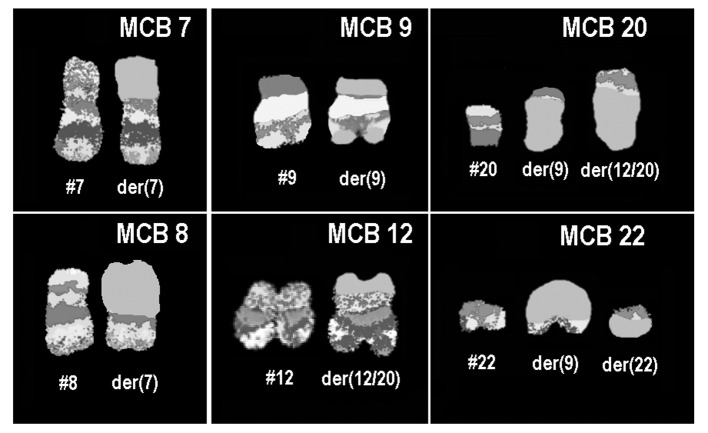
aMCB was performed to determine which chromosomes were involved in the complex rearrangement. Images demonstrate the results of MCB analysis using probe sets for chromosomes 7, 8, 9, 12, 20 and 22. Normal chromosomes are presented in the left side of each image and the derivative of the six chromosomes on the right side of normal chromosomes. MCB-probe unstained regions on the derivative chromosomes are presented in gray. #, chromosome; der, derivative chromosome; aMCB, array-proven multicolor banding.

Karyotyping was performed prior to and following chemotherapy treatment. Prior to chemotherapy, the karytype was identified as 46,XX,t(9;22)[20] and following chemotherapy was 45,XX,der(7)t(7;8),-8,der(9)t(20;9;22),-12,der(12)(12;20),+der(22)t(9;22)×2[13]/45,XX,der(7)t(7;8),-8,t(9;22) [7] (Fig. 1A). Number of cells is provided in square brackets. Dual-color FISH using a probe specific for BCR, ABL and ASS genes revealed two typical Ph chromosomes with the BCR/ABL fusion gene on the der(22). On the der(9), ABL and ASS genes at 9q34 were deleted and the BCR gene was present (Fig. 2). Chromosomes 7, 8, 9, 12, 20 and 22 were observed using WCP and/or CEP probes (data not shown). RT-PCR confirmed the presence of the BCR-ABL fusion (b3a2 transcript) revealing a major M-BCR transcript, most often identified in CML (data not shown). Finally, aMCB using probes for the corresponding chromosomes was performed as previously reported (7) (Fig. 3). The following final karyotype was determined: 45,XX,der(7)t(7;8)(p11.2;q11.2),-8,der(9) (20qter→20q11.2::9p21→9q34::22q11.2→22qter),-12,der(12) (20pter→20q11.2::12p13.3→12q24.3::12q24.3→12q15∼21.1), +der(22)t(9;22)(q34;q11.2)×2[13]/45,XX, der(7)t(7;8) (p11.2;q11.2),-8,t(9;22)(q34;q11.2)[7]. Number of cells is provided in square brackets.

Immunophenotypic analysis of peripheral blood demonstrated that the abnormal cell population positivity reacted with antibodies against CD45 (95%), HLADr (79%), CD19 (73%), CD34 (29%), CD10 (78%), CD33 (41%), CD18 (70%), CD32 (70%), CD22 (40%), CD123 (61%), CD20 (40%), CD235a (63%), CD117 (30%), CD38 (59%) and CD15 (60%). The cell population negativity reacted with additional antibodies used. Expression profiles of multilineages indicated that the patient had biphenotypic leukemia (10).

DNA sequencing of the BCR/ABL kinase domain identified no mutations.

## Discussion

In the present study, a novel case of Ph chromosome-positive CML in BC with a four-way rearrangement was observed, including five chromosomal regions, 9p21, 9q34, 12p13.3, 20q11.2, 22q11.2, an unbalanced translocation t(7;8) (p11.2;q11.2), deletions of ABL and ASS genes on der(9), monosomies 8, 12 and an additional Ph chromosome. To the best of our knowledge, these chromosomal aberrations, particularly t(7;8)(p11.2;q11.2) have not been previously observed in CML (11).

Four-way Ph translocation is extremely rare and only anecdotal cases have been described in the imatinib era. In the most recent study of CML, only 3/500 patients receiving imatinib mesylate as a frontline therapy were observed to have a four-way translocation (3).

The mechanism of development of this complex rearrangement may include a primary standard t(9;22), followed by a subsequent three-way translocation affecting chromosomes 12, 20 and the der(9). The fusion BCR/ABL signal was identified on der(22) and chromosome 22 had not rearranged with chromosome 20 or 12. These observations are consistent with a common two-step rearrangement process (12). Therefore, an inherent implication of the two-step mechanism is that variant translocations may be associated with a poorer prognosis (13).

Resistance to chemotherapy occurs as a result of increased expression of the BCR-ABL kinase from genomic amplification, clonal chromosomal evolution or mutations in the ABL kinase of the BCR-ABL gene, affecting drug interaction or kinase activity (14).

Submicroscopic ASS gene deletions in fused chromosome 9 were previously reported to be important for development of shortened CP and decreased overall survival, associated with a poor prognosis and response to interferon and imatinib mesylate (15,16).

Leukemias of ambiguous lineage are uncommon, representing ∼4% of all acute leukemias, and frequently demonstrate an aggressive disease course, with mean survival rates less than those of leukemias derived from a single-cell lineage (17). No single chromosome abnormality is unique to biphenotypic leukemia (18). In the present study, a complex cytogenetic abnormality was identified using conventional and molecular cytogenetics. Therefore, we hypothesize that leukemias of ambiguous lineage associated with cytogenetic abnormalities indicate a poorer prognosis than those without demonstrable abnormalities.

Recurrent chromosomal deletions identified in sporadic types of cancer often contain tumor suppressor genes (TSGs). TSGs function in signaling networks that protect against tumor initiation and progression and are inactivated by deletions, point mutations or promoter hypermethylation (19). For example, TCR β (7p15) (20); DLC-1 (8p21.3–22), FEZ1 (8p22) and LTPS (8p23) (21); p16INK4a, p14ARF and p15INK4b (9p21) (22); and the leukemogenesis-relevent ETV6 gene (12p)(23).

In conclusion, the present study reports a novel case of a Ph chromosome-positive CML in BC with a four-way Ph trans-location. The translocation is likely to result from a two-step mechanism. In addition to an unbalanced translocation der(7) t(7;8)(p11.2;q11.2), multiple partial chromosomal regions were deleted, partial monosomies 8, 12 and an additional Ph chromosome were identified. Immunophenotyping indicated that the patient had biphenotypic leukemia. These observations represent an adverse prognosis in CML. The patient died under treatment one month after diagnosis.
